# Differentiating Apical and Basal Left Ventricular Aneurysms Using Sphericity Index: A Clinical Study

**DOI:** 10.3390/medicina61010068

**Published:** 2025-01-03

**Authors:** Slobodan Tomić, Stefan Veljković, Armin Šljivo, Dragana Radoičić, Goran Lončar, Milovan Bojić

**Affiliations:** 1 Cardiovascular Institute ‘’Dedinje’’, 11040 Belgrade, Serbia; bobantomic99@gmail.com (S.T.); dragana.kastratovic@yahoo.com (D.R.); loncar_goran@yahoo.com (G.L.); dedinje@ikvbd.com (M.B.); 2 Faculty of Medicine, University of Banja Luka, 78000 Banja Luka, Bosnia and Herzegovina; 3 Department of Cardiosurgery, Clinical Center of University of Sarajevo, 71000 Sarajevo, Bosnia and Herzegovina; 4 Faculty of Medicine, University of Belgrade, 11000 Belgrade, Serbia

**Keywords:** left ventricular aneurysm, sphericity index, echocardiography, mitral regurgitation, surgical ventricular reconstruction

## Abstract

*Background and Objectives*: Left ventricular aneurysm (LVA) causes geometric changes, including reduced systolic function and a more spherical shape, which is quantified by the sphericity index (SI), the ratio of the short to long axis in the apical four-chamber view. This study aimed to assess SI’s value in A-LVA and B-LVA, identify influencing factors, and evaluate its clinical relevance. *Materials and Methods*: This clinical study included 54 patients with post-infarction LVA and used echocardiography to determine LVA locations (A-LVA near the apex and B-LVA in the basal segments), with SI and other echocardiographic measures assessed in both systole and diastole for the entire cohort and stratified by A-LVA and B-LVA groups. *Results*: Among the 54 patients, 41 had A-LVA and 13 had B-LVA. The mean SI was 0.55 in diastole and 0.47 in systole for the cohort. Patients with A-LVA had a mean SI of 0.51 in diastole and 0.44 in systole, while B-LVA patients exhibited significantly higher SI values, with 0.65 in diastole and 0.57 in systole, due to lower long-axis (L) values in both phases. The mean left ventricular ejection fraction (EF) was 23.95% in A-LVA and 30.85% in B-LVA, with no significant difference. However, apical aneurysms were larger (greater LVAV and LVAA) and more significantly reduced functional myocardium. LVEDV, LVESV, LVEDA, and LVESA did not differ significantly between A-LVA and B-LVA. In cases of severe mitral regurgitation (MR), SI was notably higher (0.75 in diastole) due to a marked reduction in the L axis. *Conclusions*: SI is key in differentiating A-LVA and B-LVA on echocardiography. B-LVA has lower volume and area values, but similar aneurysm and left ventricular volumes and EF. Higher SI in B-LVA is due to a reduced L-axis, and is worsened by severe mitral regurgitation (MR). Surgical ventricular reconstruction (SVR) compensates for L-axis reduction, with preservation of the L axis critical for achieving a more physiological shape. SI thus serves as a marker for left ventricular geometry and surgical outcomes.

## 1. Introduction

Post-infarction remodeling of the left ventricle (LV) is a progressive pathological process influenced by neurohormonal, mechanical, and genetic factors and characterized by structural changes in the myocardium; alterations in intracavitary volumes; and modifications in the size, shape, and function of the LV [1,2,3]. These changes, beginning with early dilation, gradually progress to ventricular dysfunction, heart failure, and increased mortality [4], underpinning the “biomechanical model” of heart failure, which suggests that its progression can eventually become independent of neurohormonal status and has inspired therapeutic strategies to disrupt the cycle of dysfunction and reverse cardiac remodeling [3,5].

On echocardiography, LV remodeling is defined as an increase in LV end-diastolic volume by 20% or more after at least six months relative to the initial value measured in the acute phase of myocardial infarction [6,7]. The size and shape of the LV contribute to dilation and heart failure through geometrical changes, with Lindbach’s anatomical observations consistently describing a more spherical LV shape during chamber remodeling (a “ball-like appearance”) [8], a concept quantified by the spherical index (SI), which reflects abnormal geometry linked to LV dilation and heart failure. Although the SI can be used in non-ischemic cardiomyopathy resulting from valvular disease or diffuse myocyte disease, the main limitation of the SI is that it analyzes the whole cavity based on a global axis ratio, as this single-plane ratio reflects linear changes in both axes across the entire chamber.

Di Donato and colleagues addressed this issue of regional shape abnormality in the LV apical region [9] by introducing a new analysis focusing on changes in the apex region. They compared normal subjects with an elliptical LV and normal SI to post-anterior infarction patients, introducing the concept of the apical conical index (CI). They also emphasized how these indices change with the development of secondary mitral insufficiency, often a complication of myocardial infarction [10,11]. An increase in the LV sphericity index (SI) is strongly correlated with more severe MR, particularly in patients with anterior ischemic heart disease. This increased sphericity may lead to posterior lateral displacement of the papillary muscles, impairing mitral valve coaptation and contributing to MR. Additionally, mid-ventricular dilation, common in anterior myocardial infarction (MI), can alter papillary muscle positioning and increase the distance between the muscles and valve leaflets, exacerbating MR. The short axis, especially in systole and diastole, also plays a critical role; dilation of the short axis is associated with more severe MR, while preserved apical conicity may indicate less severe distortion. These geometric changes help guide surgical decisions, as they determine the feasibility of valve repair versus the need for valve replacement [10,11]. Furthermore, the same authors [12] objectively classified three types of left ventricular (LV) shape abnormalities—Type I (true aneurysm), Type II (intermediate form), and Type III (ischemic dilated cardiomyopathy)—following myocardial infarction, finding that posterior infarction was more frequently associated with Type III (24% vs. 5% for Type I) and a higher incidence of mitral regurgitation compared to anterior infarction.

Myocardial infarction results in a spectrum of LV shape abnormalities related to the extent of myocardial damage, infarct location, “border zone”, severity of remodeling, nonischemic extension, and the presence of mitral regurgitation (MR) [13,14,15,16,17,18,19]. The SI correlates significantly with the degree of MR and differs significantly from normal only in patients with anterior ischemia and MR. In contrast, MR was absent in patients with a dilated ventricle but without increased global sphericity. These findings suggest that geometric changes in sphericity can potentially displace the papillary muscles posterolaterally and reduce leaflet coaptation, as previously described in circumflex artery infarction [20]. For example, LV length did not differ between patients with and without MR, but short-axis dimensions did. Conversely, the apical conical index does not disrupt the short axis (higher CI) and negatively correlates with mitral geometric parameters (tenting area and leaflet coaptation height). This indicates preserved mitral geometry when the apex is dilated, but the short axis is not similarly dilated. In patients with anterior ischemic cardiomyopathy, the anterior conical index shows a weak but inverse correlation with the tenting area and mitral coaptation point. Conversely, the SI shows a weak direct correlation with the degree of MR and papillary muscle displacement [9].

This study aims to determine the values of the SI of the LV in the presence of A-LVA and B-LVA by using echocardiography. It also aims to identify parameters that may influence SI and assess their clinical significance in daily practice: LVAVd and LVAVs, LVAAd and LVAAs, LVEDV, LVESV, LVEDA and LVESA, degree of MR, and LV EF.

## 2. Materials and Methods

This observational clinical study enrolled patients with post-infarction left ventricular aneurysms (LVAs) who presented to the Cardiovascular Institute “Dedinje” in Belgrade, Serbia, for routine transthoracic echocardiographic (TTE) assessment. Inclusion criteria included: (i) patients with the diagnosis of post-infarction LVA, (ii) patients aged 18 years and older, and (iii) patients providing informed consent to participate in the study. All patients in this study received percutaneous coronary intervention (pPCI) as part of their treatment for myocardial infarction. No surgical interventions were performed during the study period. The study received ethical approval from the Bioethical Committee of the Institute “Dedinje”, ensuring adherence to ethical guidelines and the principles of the Helsinki Declaration, with a focus on safeguarding patient rights, privacy, and confidentiality throughout the research.

### 2.1. Data Collection and Patients’ Classification

Patients underwent TTE in the left lateral decubitus position following a standardized protocol [21]. Apical aneurysms affected the apex of the LV entirely, encompassing apical segments of the septum, lateral wall, inferior, and anterior walls, or at least two of these four apical segments. Basal aneurysms involved basal segments of the inferior and posterior walls or their combination, or the basal segment of the lateral wall.

The long axis (L) of the left ventricle was measured from the 4CH view, from the apex to the midpoint of the mitral valve, while the short axis (S) was measured as the perpendicular dimension intersecting the midpoint of the long axis. The SI was calculated as the short/long axis (S/L) ratio in diastole (SId) and systole (SIs).

The volume of the left ventricular aneurysm (LVAV) was measured in systole (LVAVs) and diastole (LVAVd) using the area–length method (ml). The aneurysm area (LVAA) was measured in systole (LVAAs) and diastole (LVAAd) in apical views and calculated by planimetry (cm^2^). End-diastolic and end-systolic volumes of the left ventricle (LVEDV and LVESV) were calculated using the area–length method (ml). End-diastolic and end-systolic areas of the left ventricle (LVEDA and LVESA) were determined by planimetry (cm^2^). LV EF was calculated using the Simpson method (%).

MR was quantified using a conventional semi-quantitative assessment of jet area distribution.

### 2.2. Statistical Analysis

Numerical data were described using classical descriptive statistics, including the mean values and measures of variability such as standard deviation. The distribution of numerical variables was tested using the Kolmogorov–Smirnov test, ensuring a normal distribution for parametric methods. Variables not meeting this criterion were analyzed with non-parametric methods. Baseline characteristics and mean values of the observed parameters were compared using the Chi-square test, Student’s *t*-test, or the Mann–Whitney test as a supplementary method. Data analysis was performed using SPSS software version 22, with a significance level of 0.05 applied to all statistical methods.

## 3. Results

The study group consisted of 54 patients with 6-month-old post-infarction left ventricular aneurysms (36 men, mean age: 63.19 years, and 18 women, mean age: 65.06 years).

Table 1 compares apical (A-LVA) and basal (B-LVA) aneurysms in terms of aneurysm volume in diastole (LVAVd) and systole (LVAVs), aneurysm area in diastole (LVAAd) and systole (LVAAs), left ventricular volume in diastole (LVEDV) and systole (LVESV), and left ventricular area in diastole (LVEDA) and systole (LVESA). Basal aneurysms demonstrated significantly lower values for LVAVd, LVAAd, and LVAAs.

When we compared the ejection fractions (EFs), left ventricular end-diastolic volumes (LVEDVs), and left ventricular end-systolic volumes (LVESVs) between patients with apical and basal LVA, we found that basal aneurysms had higher EFs (30.85 ± 14.51 vs. 23.95 ± 11.16, *p* = 0.770) and LVEDVs (290.54 ± 136.38 mL vs. 264.61 ± 96.87 mL, *p* = 0.451), while LVESV was slightly lower in the basal group (212.31 ± 126.81 mL vs. 216.59 ± 183.16 mL, *p* = 0.938). However, none of the differences were statistically significant.

Table 2 shows the mean, minimum, and maximum values of the SI in systole and diastole. SI values were higher in diastole.

Table 3 illustrates SI values for apical (A-LVA) and basal (B-LVA) aneurysms. Significant differences in SI were observed, with higher SI in both diastole and systole for basal aneurysms, attributed to a shorter long axis (L) in basal aneurysms.

Table 4 compares the long-axis (L) and short-axis (S) dimensions in diastole (Ld, Sd) and systole (Ls, Ss) between apical and basal aneurysms. Basal aneurysms exhibited significantly shorter L dimensions in both diastole and systole.

Table 5 examines the impact of severe MR on LV dimensions. Severe MR was associated with a significant reduction in the long axis (L) dimensions in both diastole and systole. The short axis in diastole (Sd) showed no significant increase, while the systolic short axis (Ss) remained largely unchanged.

Table 6 outlines the diastolic sphericity index (SId) relative to MR severity. A significant increase in SId was observed in cases of severe MR.

Table 7 provides systolic sphericity index (SIs) values based on MR severity. No significant changes in SIs were observed with severe MR.

## 4. Discussion

In our study, the majority of LVA were located at the apex, consistent with findings from similar research [22,23,24]. Basal aneurysms, although less common, were significantly smaller than apical aneurysms, as evidenced by their lower diastolic volumes and both diastolic and systolic surface areas. These findings indicate that basal aneurysms generally involve less extensive remodeling of the ventricular wall compared to apical aneurysms. Additionally, the sizes of the aneurysms observed in our cohort were slightly larger than those reported in previous studies, potentially reflecting variations in patient populations or diagnostic methodologies [25]. The EF was markedly reduced in both apical and basal left ventricular aneurysms, with greater impairment observed in apical cases, aligning with existing literature [24,26,27]. Furthermore, the study highlighted significant differences in the SI between A-LVA and B-LVA. Basal aneurysms exhibited higher SI values in both diastole and systole, driven by shorter long-axis dimensions. Severe MR was associated with an increased diastolic SI, while systolic SI remained unchanged. These findings underscore the geometric distinctions between apical and basal aneurysms, with basal aneurysms presenting a more spherical geometry, particularly in cases of severe MR.

This SI reflects global ventricular changes, using one plane to analyze the ratio of the short to long axes and comparing the chamber volume with the theoretical sphere’s volume (defined by measuring the long axis as the sphere’s diameter). Changes in the size and shape of the left ventricle from a geometric perspective can lead to dilation and heart failure. Linzbach’s anatomical observations describe a consistent finding of a more spherical chamber shape with increasing size, indicative of abnormal chamber-remodeling geometry [8]. This concept involves increased ventricular sphericity (“ball-like appearance”) expressed by the SI, a parameter of abnormal geometry changes associated with heart failure in left ventricular dilation [9].

Di Donato et al. [9] conducted a comparative analysis of ventricular geometry, focusing on the size and shape of the left ventricle in normal individuals and those with ischemic anterior dilated cardiomyopathy. Their findings revealed that the geometry of the left ventricle undergoes significant alterations following chronic anterior myocardial infarction. In patients without mitral regurgitation, both the long and short axes of the ventricle increase proportionally, with a higher SI observed only in cases where mitral regurgitation occurs, attributed to increased septum-to-lateral wall dimensions. Additionally, the study introduced a novel parameter, the apical conicity index, to quantify specific abnormalities in the apical shape of the left ventricle caused by anterior infarction, independent of changes in the SI. Although Di Donato et al. [9] document significant changes in ventricular size and geometry (e.g., larger volumes, increased longitudinal and transverse diameters, greater tenting area of the mitral valve, and leaflet coaptation height) in ischemic cardiomyopathy post-chronic anterior infarction, the SI consistently differs from that of a normal heart. The long and short axes proportionally increase, maintaining a constant ratio except in cases where anterior infarction is associated with mitral regurgitation. These observations have shifted focus to regional shape abnormalities as an index of anterior infarction. At the same time, secondary late mitral regurgitation globally affects the ventricle, altering the SI. A higher SI occurs only when the chamber globally enlarges, coinciding with secondary mitral regurgitation. This valvular complication of acute myocardial infarction [11] stretches distant myocardial regions and increases global sphericity [9].

Di Donato et al. [9] found that the SI in normal populations was 0.52 in diastole and 0.45 in systole. In patients with anterior infarction, the SI was 0.53 in diastole and 0.46 in systole. In true apical aneurysms, the SI was 0.55 in diastole and 0.45 in systole. Marsan et al. reported an average left ventricular SI of 0.51 using 2D echocardiography [13].

Our results showed that the overall SI was 0.55 in diastole and 0.47 in systole, aligning with the study by Di Donato et al. [9]. Statistically significant differences were observed between apical and basal aneurysms. For apical aneurysms, the SI was 0.51 in diastole and 0.44 in systole. The values were significantly higher for basal aneurysms: 0.65 in diastole and 0.57 in systole. These results are attributed to the lower long-axis (L) value in basal aneurysms. Specifically, the L axis in patients with B-LVA aneurysms approximated the physiological range (Ld 85.62 mm, Ls 80 mm) compared to significantly higher values in A-LVA aneurysms (Ld 97.27 mm, Ls 96.54 mm).

As previously mentioned, the conical index (CI) negatively correlated with mitral valve geometry parameters (tenting area and leaflet coaptation height) when only the apex was dilated, and the short axis was not dilated to the same extent. These observations reflect the differences between apical and global dilatation in patients with ischemic cardiomyopathy. Mitral function was better (lower tenting area and coaptation height) when the apex was disproportionately dilated compared to the short axis (larger conical index). Conversely, mitral function was impaired (greater distance between papillary muscles and higher degree of mitral regurgitation) when the SI was high [9].

In a subgroup of patients with mitral regurgitation (MR), the SI was higher than in a normal chamber, measured at 0.58 in diastole and 0.51 in systole, while the apical conical index did not correlate with MR. Left ventricular volumes did not significantly differ in the presence or absence of MR [9].

In our study, the conclusions regarding the impact of MR on SI align with Di Donato’s findings. Specifically, the greater the MR, the higher the SI. The key difference lies in the mechanism of the SI increase. Di Donato attributed this to an increase in the short axis (patients with anterior infarction and chamber dilation). Our results indicate that the long axis (L) statistically significantly decreases in diastole and systole as MR severity increases, thus raising the SI.

Geometric changes in sphericity may arise primarily (e.g., in cases involving the formation of a basal aneurysm) or secondarily due to dilation of distant myocardium, potentially disrupting the relationship between the papillary muscles, which can contribute to MR. The altered architecture of the left ventricle’s shape in the presence of a basal aneurysm is reflected by an increased SI (0.65) compared to an apical aneurysm (0.51), which is approximately the SI in a normal heart. Mild to moderate MR does not significantly raise SI, while severe MR markedly increases SI (e.g., 0.75 in diastole compared to 0.65 in B-LVA aneurysms). Severe MR combined with a basal aneurysm significantly elevates SI values compared to A-LVA aneurysms and the normal population.

The normal left ventricular shape is defined as elongated and ellipsoid. This ellipsoid shape is vital for optimal function. Pathological conditions such as ischemic heart disease, valvular disease, and cardiomyopathies result in the loss of this shape, leading to ventricular dilation and increased sphericity. The transition from an ellipsoid to a globular (spherical) shape with disease indicates the onset of left ventricular dysfunction and triggers the progression of heart failure. In advanced heart failure caused by post-infarction left ventricular aneurysm, reducing chamber volume through surgical restoration can help reestablish ventricular geometry, leading to beneficial clinical outcomes [28].

All reports highlight that the goal of SVR is to reshape the left ventricle into a more ellipsoid form. However, no studies demonstrate that the left ventricle becomes less spherical (i.e., more ellipsoid) postoperatively. Preoperatively, the left ventricle is not necessarily more spherical than normal (unless MR is present), as elongation is proportionate to width increase following anterior myocardial infarction [29].

After SVR, the left ventricle does not become more ellipsoid because surgical length reduction (L-axis) often exceeds width reduction (S-axis), depending on the use of conical devices for reshaping. In cases involving B-LVA aneurysms, it is essential to preserve the L-axis length within the physiological range. For A-LVA aneurysms, the L-axis is reduced during SVR, resulting in a more ellipsoid and less spherical shape. In contrast, for B-LVA aneurysms, where L is closer to normal values, eliminating the aneurysm through SVR leaves the heart’s configuration more spherical (higher SI) and less ellipsoid.

With significant MR, patients with B-LVA aneurysms exhibit distinctly more spherical heart configurations. The SI in basal aneurysms can serve as a marker of not only the shape and size of the left ventricle, but also the adequacy of surgical reconstruction.

Recent advancements in 3D echocardiography have enhanced the evaluation of left ventricular geometry and function in ventricular aneurysms. Unlike 2D imaging, 3D echocardiography provides superior volumetric assessment and detailed visualization of complex ventricular shapes. This improves the delineation of left ventricular remodeling, offering more precise insights into MR pathophysiology and aiding in the assessment of MR severity, thus refining clinical decision making [30].

Additionally, strain imaging via speckle-tracking echocardiography is a crucial tool for assessing myocardial function by quantifying myocardial deformation. It complements geometric measurements like SI and is particularly effective in detecting early myocardial dysfunction and evaluating ischemic or infarcted areas. While 3D echocardiography provides detailed anatomical insights into ventricular geometry, strain imaging focuses on myocardial function, identifying abnormalities in regions with minimal geometric changes. This combined approach enhances the assessment of cardiac health by evaluating both ventricular structure and myocardial performance [31].

Cardiac magnetic resonance (CMR) is another powerful imaging modality, particularly for assessing aneurysm size, location, and myocardial viability. CMR provides high-resolution imaging, allowing for precise measurement of aneurysm dimensions and a better understanding of myocardial scar tissue. This modality is especially valuable in complex cases, where other imaging techniques may not offer sufficient detail. Furthermore, CMR plays a crucial role in evaluating myocardial viability, a critical factor in determining the potential for recovery and guiding surgical decisions. Unlike echocardiography, CMR offers a detailed assessment of both the size and functional status of the aneurysm, aiding in the comprehensive evaluation of ventricular function [32].

This study has several limitations. The small sample size may limit the detection of significant differences in aneurysm characteristics and impact the generalizability of the findings. Additionally, the single-center design restricts the representativeness of the results, and a multi-center study would better reflect diverse clinical practices and patient demographics. Finally, the specific clinical setting and patient population may reduce the applicability of the findings to other healthcare environments.

## 5. Conclusions

The SI values are statistically higher in the presence of a basal aneurysm compared to an apical aneurysm due to the reduced long axis (L) of the left ventricle in basal aneurysms. Although basal aneurysms have statistically lower volume and surface area values than apical aneurysms, the overall left ventricular volume, surface area, and EF are comparable between the two types. Severe MR significantly increases SI, primarily due to a marked reduction in the long axis, particularly in diastole, in the presence of basal aneurysms.

## Figures and Tables

**Table 1:** Results for aneurysm volume in diastole (LVAVd) and systole (LVAVs), aneurysm area in diastole (LVAAd) and systole (LVAAs), left ventricular volume in diastole (LVEDV) and systole (LVESV), and left ventricular area in diastole (LVEDA) and systole (LVESA) for apical (A-LVA) and basal (B-LVA) aneurysms.

	A-LVA (41)	B-LVA (13)	*p*
LVAVd	84.75 ± 58.82	40.38 ± 44.76	0.016
LVAVs	74.48 ± 58.45	45.57 ± 61.63	0.131
LVAAd	22.70 ± 9.80	13.36 ± 8.73	0.003
LVAAs	20.64 ± 10.12	13.53 ± 9.28	0.029
LVEDV	264.61 ± 96.87	290.54 ± 136.38	0.451
LVESV	216.59 ± 183.16	212.31 ± 183.16	0.938
LVEDA	54.64 ± 13.58	56.49 ± 18.37	0.698
LVESA	45.64 ± 13.04	46.03 ± 18.13	0.931

**Table 2:** Sphericity index values in diastole (SId) and systole (SIs).

	SId (54)	SIs (54)
X ± SD	0.55 ± 0.12	0.47 ± 0.14
min	0.40	0.28
max	1.25	1.30

**Table 3:** Sphericity index values in diastole (SId) and systole (SIs) for apical (A-LVA) and basal (B-LVA) aneurysms.

SI	A-LVA (41)	B-LVA (13)	*p*
SId	0.51 ± 0.07	0.65 ± 0.19	0.001
SIs	0.44 ± 0.07	0.57 ± 0.23	0.002

**Table 4:** Long- (L) and short (S)-axis dimensions in diastole (Ld, Sd) and systole (Ls, Ss) for apical (A-LVA) and basal (B-LVA) aneurysms.

	A-LVA (41)	B-LVA (13)	*p*
Ld	97.27 ± 14.96	85.62 ± 12.46	0.014
Ls	96.54 ± 13.39	80.00 ± 11.42	0.001
Sd	50.49 ± 7.74	52.46 ± 6.57	0.412
Ss	42.07 ± 8.75	42.85 ± 7.90	0.778

**Table 5:** Long-axis (Ld, Ls) and short-axis (Sd, Ss) dimensions in relation to mitral regurgitation severity (MR).

N (28)	MR	Ld (mm)	Ls (mm)	Sd (mm)	Ss (mm)
	0.50	102	97	46	44
	1.00	99	97.58	53.33	45.3
	1.50	117	110	50	40.5
	2.00	93	90.33	51.89	43
	3.00	75.25	71	50	42.47
*p*		0.010	0.011	0.879	0.951

**Table 6:** Diastolic sphericity index (SId) values relative to MR severity.

N	MR	SId (X ± SD)	min	max
1	0.50	0.45		
12	1.00	0.53 ± 0.08	0.45	0.61
2	1.50	0.43 ± 0.04	0.22	0.63
9	2.00	0.55 ± 0.07	0.46	0.65
4	3.00	0.75 ± 0.32	0.64	0.92
Ʃ28		0.56 ± 0.16		
*p*		0.027		

**Table 7:** Systolic sphericity index (SIs) values relative to MR severity.

N	MR	SIs (X ± SD)	min	max
1	0.50	0.45		
12	1.00	0.45 ± 0.04	0.36	0.55
2	1.50	0.36 ± 0.11	0.12	0.60
9	2.00	0.48 ± 0.05	0.37	0.59
4	3.00	0.73 ± 0.08	0.56	0.89
Ʃ28		0.49 ± 0.14		
*p*		0.063		

## Data Availability

Data are available upon request.
